# Integrative transcriptomic and metabonomic profiling analyses reveal the molecular mechanism of Chinese traditional medicine huankuile suspension on TNBS-induced ulcerative colitis

**DOI:** 10.18632/aging.202427

**Published:** 2021-02-01

**Authors:** Zhenglan Han, Hanyan Wang, Dongmei Guo, Jingping Zhang

**Affiliations:** 1 Department of Biochemistry, School of Preclinical Medicine, North Sichuan Medical College, Nanchong 637100, Sichuan Province, China

**Keywords:** ulcerative colitis, Huankuile suspension, traditional Chinese medicine, transcriptomic, metabonomic

## Abstract

This study aimed to investigate the therapeutic mechanism of Huankuile suspension (HKL), a typical traditional Chinese medicine, on ulcerative colitis (UC) in a rat model. UC model was established by 2,4,6-trinitrobenzene sulfonic acid (TNBS) enema. Then, the rats were randomly divided into three groups: water treated group, HKL treated group and 5- amino salicylic acid (5-ASA) treated group. After 7 days treatment, the histological score in the HKL treated group was comparable with those in the control group. qRT-PCR and western blot demonstrated that HKL could significantly decreased pro-inflammatory cytokines, including *TNF-α*, *IL-1β* and *IL-6*, while having less effect on anti-inflammatory cytokines, including *IL-4* and *IL-10*. Transcriptomic analysis identified 670 differentially expressed genes (DEGs) between HKL treated UC rats and water treated UC rats. These DEGs were mostly related with immune response. Besides, metabonomic profile revealed 136 differential metabolites which were significantly enriched in “pyrimidine metabolism”, “glutathione metabolism”, “purine metabolism” and “citrate cycle”. Finally, integrated analysis revealed that metabonomic pathways including “steroid hormone biosynthesis”, “pyrimidine metabolism”, “purine metabolism”, and “glutathione metabolism” were altered by HKL at both transcriptomic and metabonomic levels. HKL could inhibit inflammation and regulate bile metabolism, pyrimidine metabolism, purine metabolism, glutathione metabolism and citrate cycle.

## INTRODUCTION

Ulcerative colitis (UC) is an inflammatory bowel disease (IBD), characterized by the damage of mucosa and submucosa of the colon. The major symptom includes blood in stool, pain, increased defection in diarrhea and tenesmus [1–3]. During the past years, the prevalence and incidence increased in developed and developing countries [4, 5]. Epidemiology study suggested that the incidence of IBD is highest in 20-30-year-old group and reaches to another peak at 60-70-year-old group [6]. The high incidence of UC brings serious economic burden and significantly decreased quality of life for both patients and their families.

Up to now, the definite pathogenesis of UC remains unclear. It is believed that the immune tolerance defect induced by loss of mucosal barrier integrity is the primary mechanism [7]. The balance between Th1 and Th2 as well as the cytotoxicity of the intestinal epithelial cells caused by interleukin (IL) were reported function in UC development. Auto-immune was also involved in the progression of UC with the present of antibodies against epithelial cells in serum and mucosal [8, 9]. In recent years, impaired homoeostatic balance between the enteric microflora and the host's mucosal immunity was also reported to be possible pathogenesis of UC [10]. In addition, living status, microbial drugs, stress and diet may also be provoking factors of UC [11–13].

Currently, there is no permanent cure for UC. The available drug therapy, including 5-aminosalicylates acid (5-ASA), corticosteroids and thiopurines, could induce clinical remission and promote healing process of colonic mucosa [14, 15]. Besides, biological drugs targeting specific pathways and non-biological agents targeting different pathways were also developed [13]. However, considering the adverse effects and hormone resistance or dependence, seeking for other drugs of UC treatment is still warranted.

The traditional Chinese medicine (TCM), derived from herbs, shows higher safety in treating disease including UC and is widely used in clinical therapy in Asian countries [16, 17]. Some TCM drugs illustrated promising therapeutic effects in treatment of UC, [18–20]. Huankuile suspension (HKL) is a new recipe, which composed of 8 kinds of herbs, including Trukish galls, Coptis chinensis, pomegranate flower, amber, tabasheer and plantain herb. However, the molecular mechanism of HKL has not been investigated previously.

Recent advances of high-throughput technologies, such as genome-wide gene expression profiling and metabolomics analysis have greatly facilitated the research on UC pathogenesis. RNA sequencing studies have identified UC specific differential expression of genes, fusion genes and mutated genes [21]. Small-molecule metabolites in biological fluids including serum, plasma, and urine were identified by metabolomics analysis to explore the metabolites related to UC [22–24]. In this study, we investigated the molecular mechanism of HKL on treating UC by integrating RNA sequencing and metabolomics analysis in a UC rat model. The disturbed transcriptomic and metabonomic profiles of HKL were characterized and the underlying molecular mechanism was elucidated.

## RESULTS

### HKL treatment significantly relief UC symptom

**Figure 1:**
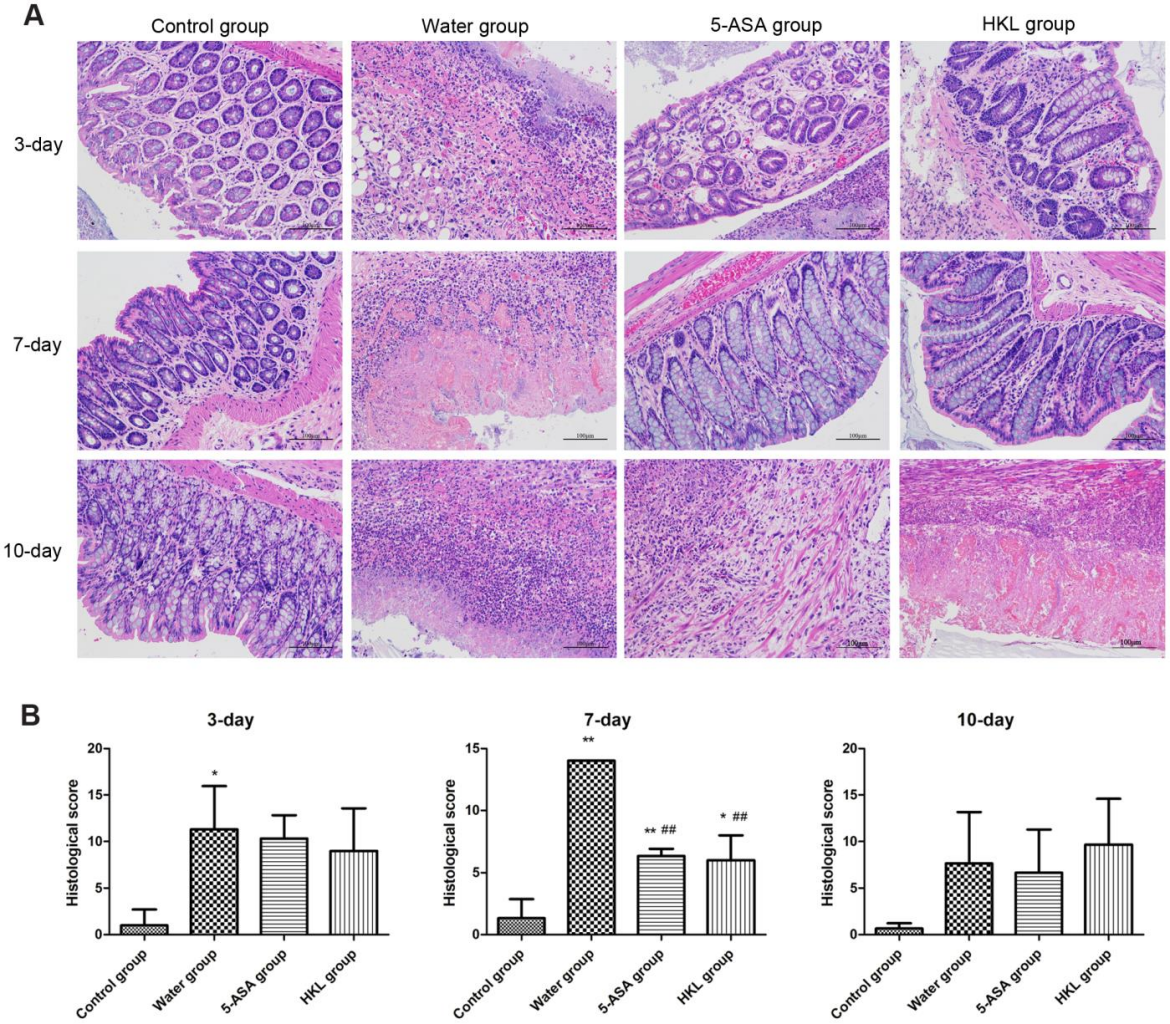
**Histological grading of colitis.** (**A**) Representative photographs showing histological score assigned to biopsies, magnification 10X. (**B**) Sum of inflammation score according to Dieleman scoring system in normal group, 5-ASA group, HKL group and water group after 3 days, 7 days and 10 days treatment (n = 3 at each time points in each group). * *P* < 0.05, ** *P* < 0.01 compared with control group, ## *P* < 0.01 compared with water group.

The 2,4,6-trinitrobenzene sulfonic acid (TNBS) administration successfully induced UC in Wistar rats, with characters of the fragmentation, shedding in intestinal mucosal epithelial cells and inflammatory infiltration in lamina propria. After treatment for 7 days, the symptoms were partly relieved in HKL and 5-ASA treated groups, which demonstrated basically intact intestinal mucosal epithelial tissues and reduced amount of inflammatory cells. In water treated group, the intestinal mucosa epithelial fragmentation, shedding, intestinal mucosal congestion and edema, and lamina propria inflammatory cell infiltration were found. These results indicated the effective function of HKL in UC treatment (Figure 1A). Histopathology scores based on inflammation, extent, regeneration, crypt damage and percent involvement were graded and calculated. We found the 5-ASA and HKL could significantly relieve the symptom after 3 days or 7 days treatment. Among all groups, the most obvious improvement of HKL was observed after 7 days treatment (Figure 1B). These results revealed that HKL indeed functioned in UC.

### HKL regulated the expression of cytokines

**Figure 2:**
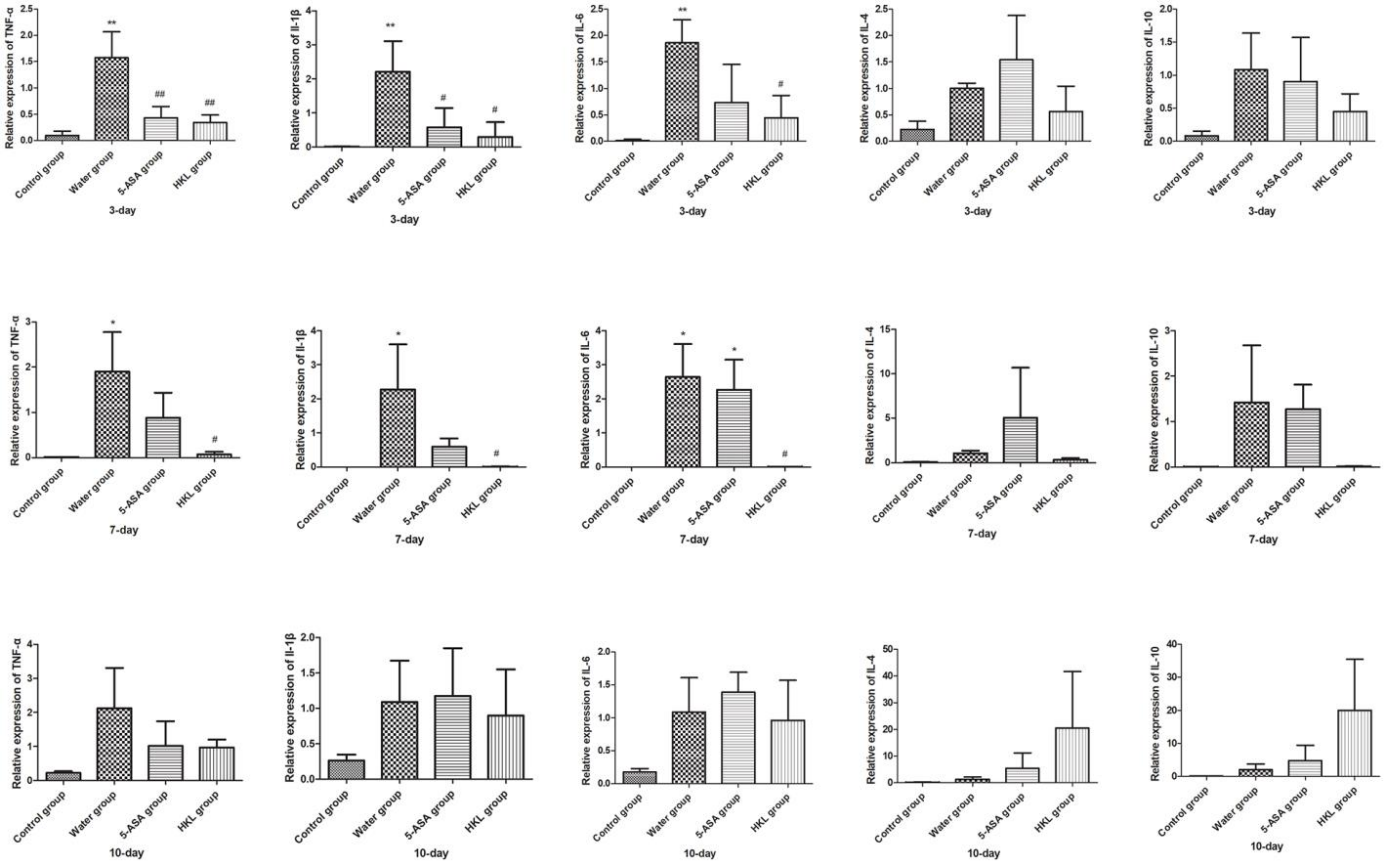
**HKL suppressed inflammatory factors expression at mRNA level.** UC rats were treated with water, 5-ASA and HKL for 3 days, 7 days and 10 days. Tissues were harvested for qRT-PCR analysis (n = 3 at each time points in each group). Each sample was tested in triplicate. *, *P* < 0.05; **, *P* < 0.01 compared with control group. # *P* < 0.05, ## *P* < 0.01 compared with water group.

**Figure 3:**
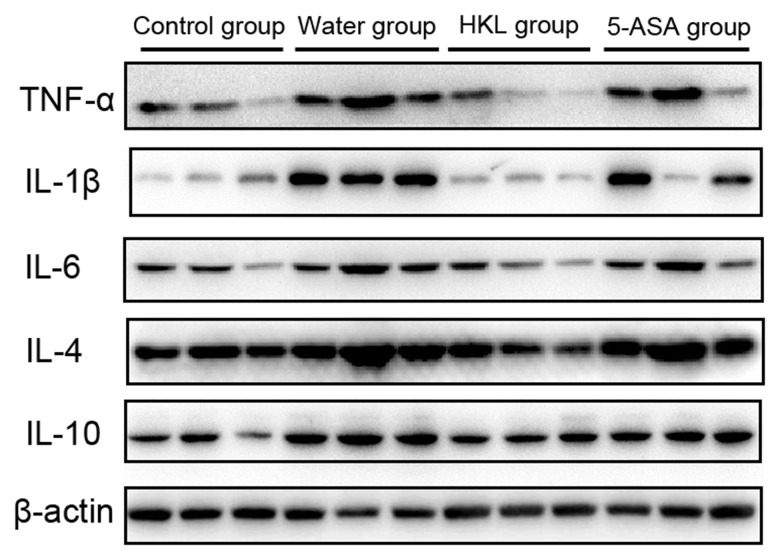
**HKL inhibited expression of inflammatory factors at protein level.** UC rats were treated with water, 5-ASA and HKL for 7 days. Colon tissues were harvested for western blot analysis. Each experiment was repeated in three samples.

Next, we sought to investigate the mechanism, through which HKL regulated the progression of UC. As the inflammation was the primary mechanism in UC, the expression of inflammation cytokines was explored. Rats were administered for 3 days, 7 days or 10 days and the expression of *TNF-α*, *IL-1β*, *IL-6*, *IL-4* and *IL-10* was determined. As shown in Figure 2, the expression of pro-inflammatory cytokines, including *TNF-α*, *IL-1β* and *IL-6* was significantly increased in water group, while treatment of 5-ASA and HKL could significantly reduce the expression levels of these pro-inflammation cytokines at 3 days and 7 days. However, the anti-inflammatory cytokines, *IL-4* and *IL-10*, were not significantly different among groups (Figure 2). These results indicated that HKL might block UC progression by inhibiting expression of pro-inflammation cytokines. The protein expression of inflammation cytokines was further confirmed by western blot. As shown in Figure 3, protein expression levels of *TNF-α*, *IL-1β* and *IL-6* were significantly decreased compared with water group, while the protein expression of anti-inflammation factors, *IL-4* and *IL-10* were not significantly different among groups (Figure 3). Taken the above results together, we proposed that HKL might function in UC by regulating expression of pro-inflammation cytokines but not anti-inflammation cytokines.

### Identification and functional analysis of DEGs

**Figure 4:**
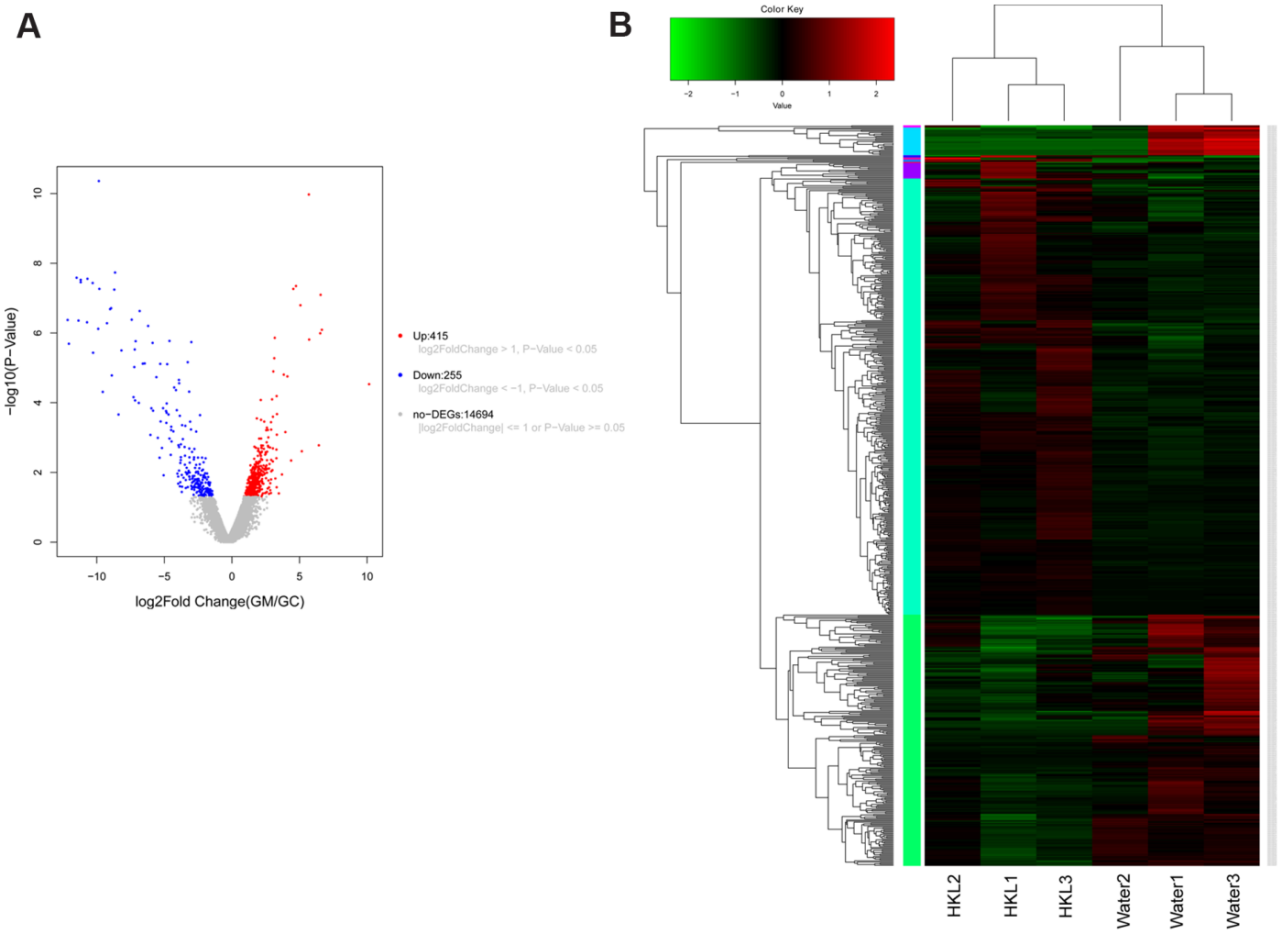
**Expression signature of differentially expressed genes (DEGs).** (**A**) Volcano plot displayed the distribution of DEGs. The blue dots represent down-regulated genes and the red dots represent up-regulated genes. (**B**) Heatmap of DEGs (n = 3 in each group). Each row represents one individual sample, and each column represents one gene. High expression levels are shown in red and low expression levels in green. UC rats were treated with water or HKL for 7 days and colon tissues were extracted for transcriptomics analysis.

**Figure 5:**
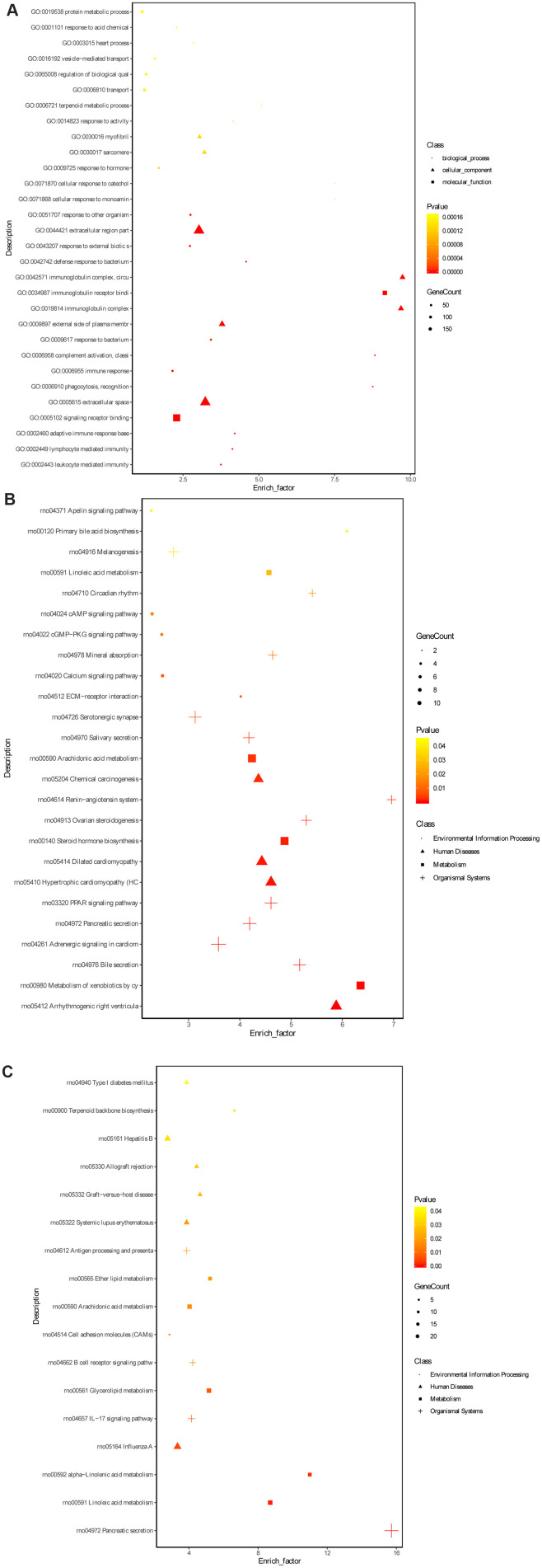
**Gene ontology terms and KEGG pathway enrichment of DEGs.** (**A**) The significantly enriched GO terms of DEGs in molecular function, biological process and cellular component. (**B**) The significantly enriched KEGG pathways of the upregulated genes. (**C**) The significantly enriched KEGG pathways of the downregulated genes. The dot represents biological process, triangle represents cellular component and square represents molecular function.

To further explore the underlying mechanism of HKL on UC progression, the transcription profile and metabolism profile of UC rats in water group and HKL group at 7-day treatment were investigated. Based on criteria of *P* < 0.05 and |log_2_ fold change (FC)| >1, a total of 670 differentially expressed genes (DEGs), including 415 up-regulated and 255 down-regulated genes, were identified between HKL group and water group (Figure 4A, Supplementary Table 1). Heatmap showed the DEGs could separate the HKL treated samples and water treated samples, indicating the DEGs are reliable (Figure 4B). Then, Gene Ontology (GO) and Kyoto encyclopedia of genes and genomes (KEGG) pathways were performed to explore the biological function of DEGs. Based on threshold of false discovery rate (FDR) < 0.05, a total of 293 GO-terms were significantly enriched (Supplementary Table 2). The most significant enriched GO terms were related with immune system, including “leukocyte mediated immunity” (n = 39, FDR = 0), “lymphocyte mediated immunity” (n = 35, FDR = 0), “adaptive immune response based on somatic recombination of immune receptors built from immunoglobulin superfamily domains” (n = 36, FDR = 0), “immune response” (n = 84, FDR = 0), and “B cell mediated immunity” (n = 30, FDR = 3.35 ×10^-10^) (Figure 5A). Besides, we performed KEGG enrichment analysis for upregulated genes and downregulated genes, respectively. As shown in Figure 5B, the KEGG pathways of “PPAR signaling pathway”, “ECM-receptor interaction”, “calcium signaling pathway”, “cGMP-PKG signaling pathway”, and “cAMP signaling pathway” were significantly activated after HKL treatment (Supplementary Table 3), while the KEGG pathways of “Complement and coagulation cascades”, “IL-17 signaling pathway”, “cell adhesion molecules (CAMs)” were significantly suppressed (Figure 5C, Supplementary Table 4).

### Protein-protein interactions (PPI) network construction

**Figure 6:**
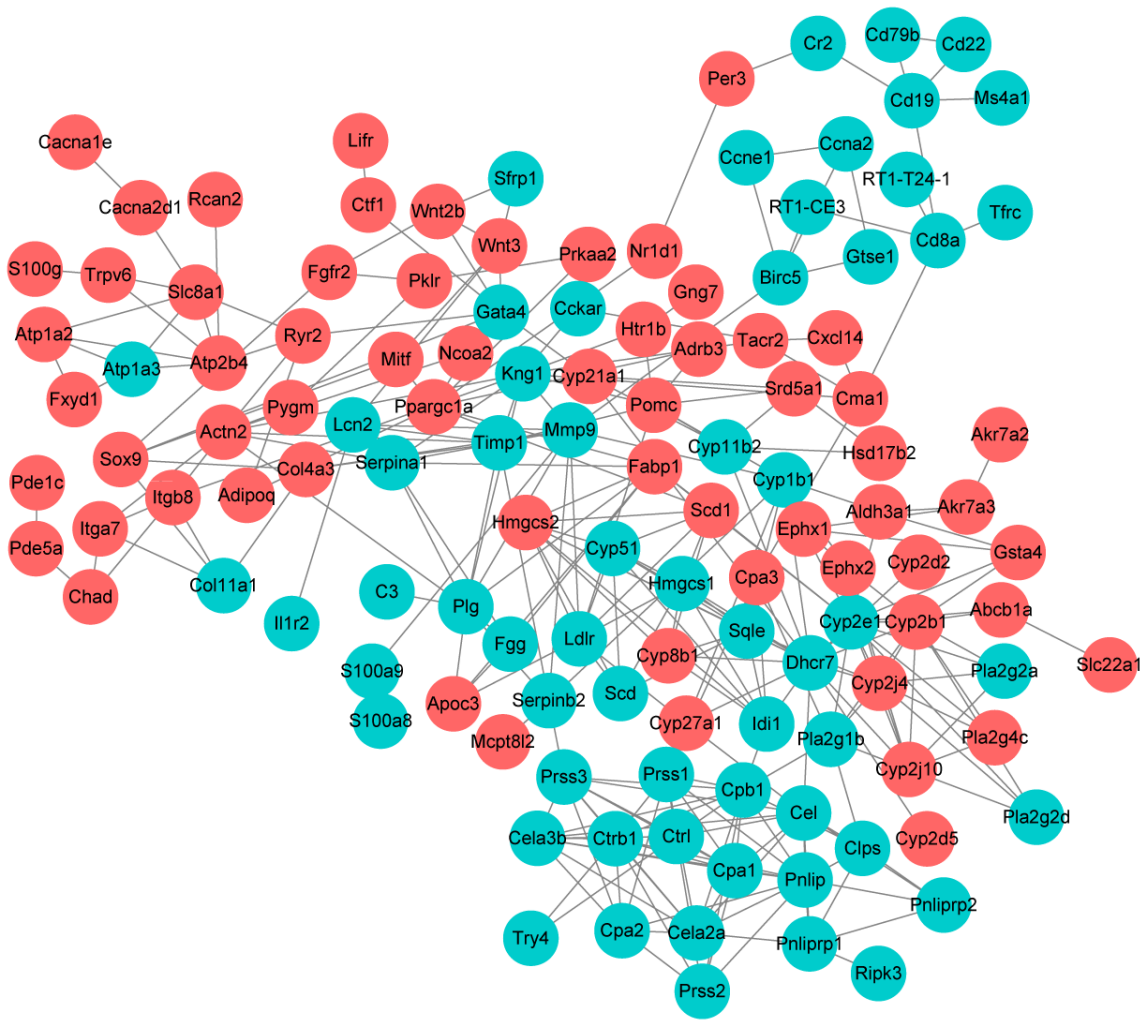
**Protein-protein interaction network construction.** Each node represents one differentially expressed protein. Each edge represents regulation. Red, up-regulated expressed protein; green, down-regulated expressed protein.

Further, we selected KEGG pathways that might be related with UC progression and built a PPI network among the DEGs enriched in these pathways. As shown in Figure 6, Dhcr7 (degree = 17), Cyp2e1 (degree = 14), Pnlip (degree = 12), Cpb1 (degree = 12) and Kng1 (degree) were the 5 nodes with the highest node degree.

### Differentially expressed metabolites identification

**Figure 7:**
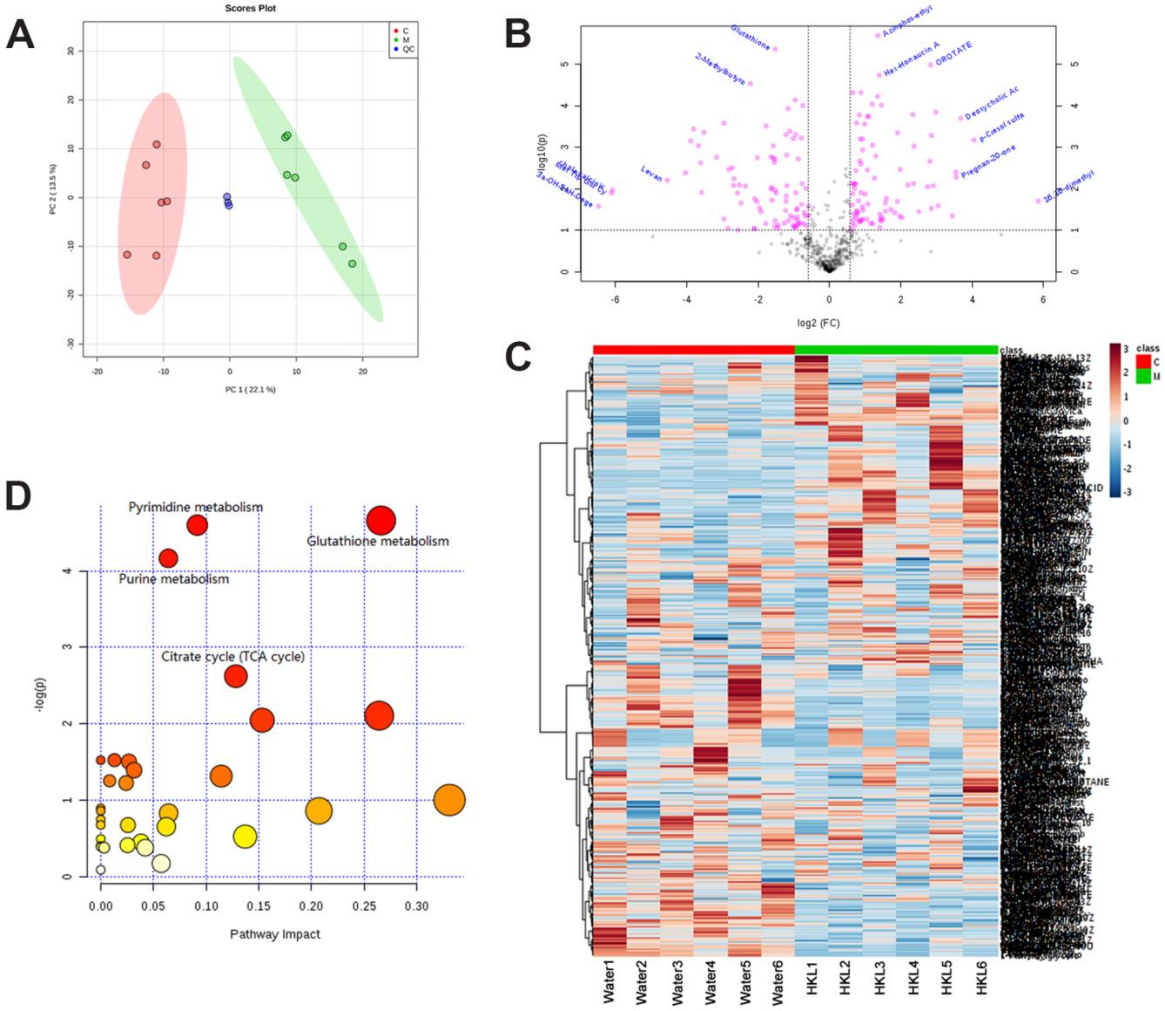
**Identification of differentially expressed metabolites.** (**A**) Principal Component Analysis examines the distribution of samples. (**B**) The log2 ratio of fold change and –log(P-values) plotted in the form of volcano plots. Red dots represent differentially expressed metabolites and black dots represent non-significantly changed metabolites. (**C**) Heatmap illustrates the metabolite profile. The column represents sample and the row represents relative molecular mass. (**D**) The bubble graph represents the significantly enriched pathways of DEGs. UC rats were treated with water or HKL for 7 days and fetal samples were collected for metabolomics assay. LC-MS/MS analyses were performed on 6 rats from water or HKL treated groups. Each bubble represents one individual pathway. The area of the bubble positively correlates with the importance in pathway. C, M, QC, quality control samples.

We further performed metabonomic analysis for HKL and water treated UC rats. Principal Component Analysis of samples showed that the samples of the same group are relatively concentrated in the two-dimensional spatial distribution and the quality control (QC) sample was distributed around the origin. This result indicated that the method was stable and had good repeatability (Figure 7A). A total of 136 differential metabolites were identified based on criteria of variable importance in projection (VIP) > 1, *P* < 0.05 and |log_2_FC| > 0.565, including 71 upregulated metabolites and 65 downregulated metabolites (Figure 7B and Supplementary Table 5). Hierarchical cluster analysis revealed obvious difference between UC rats in water group and HKL group, indicating the high reliability of metabonomic analysis (Figure 7C). KEGG pathway analysis indicated pyrimidine metabolism, glutathione metabolism, purine metabolism and citrate cycle were four of the most significantly enriched pathways (Figure 7D).

### Integrative transcriptomic and metabonomic molecular profiling analysis

**Figure 8:**
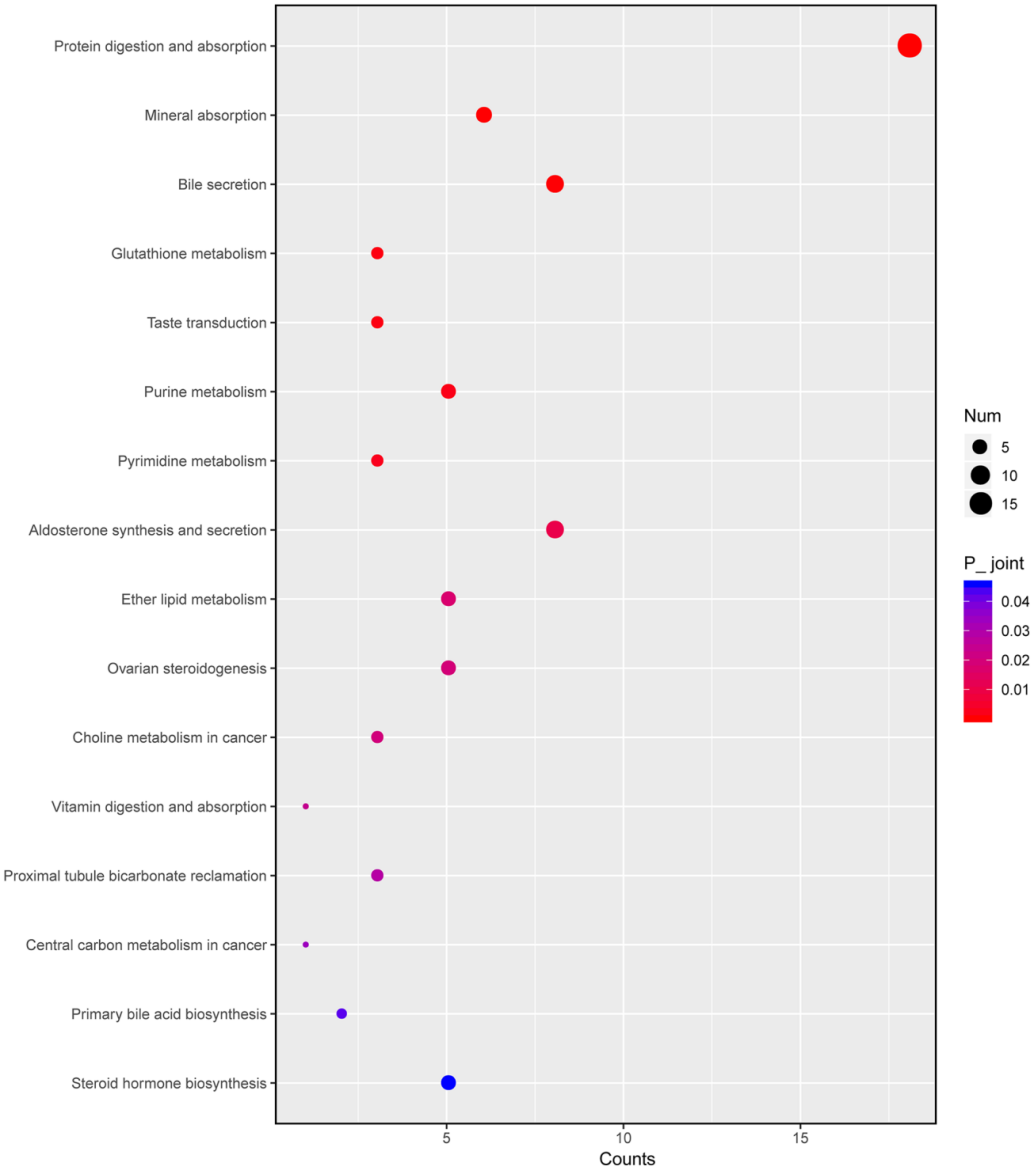
**Integrated altered metabolic pathways in HKL treated UC rats according to our transcriptomic and metabolomics data.** The bubble graph represents the enriched DEGs. The column represents enriched counts; the row represents enriched pathway. Each bubble represents one individual pathway. Different colors represent significance P value.

Next, we integrated transcriptomic and metabonomic data to further explore the pharmacological effect of HKL. The KEGG pathways being disturbed at both transcriptomic level and metabonomic level were identified. The pathways of “steroid hormone biosynthesis”, “primary bile acid biosynthesis”, “central carbon metabolism in cancer”, “vitamin digestion and absorption”, “pyrimidine metabolism”, “purine metabolism”, and “glutathione metabolism” were significantly enriched (Figure 8).

## DISCUSSION

In this study, the molecular mechanism of HKL on UC was explored. We demonstrated that intrarectal administration of HKL could decrease the inflammation of the colon, relieve the symptom and promote repair by down-regulating the pro-inflammatory cytokines. To further explore the underlying chemical composition and therapeutic targets of herbal medicines, transcriptomic and metabolomics changes were characterized in a parallel and integrative manner, providing a comprehensive molecular profile.

UC is the chronic inflammatory condition of mucosa with large intestine in the rectum and colon, which involves the immune response resulting in epithelial barrier breach of mucosa, immune cells migration across the endothelial layer and the release of mediators [13]. Active inflammatory cells could up-regulate the cytokines and these cytokines could positively feedback, which exacerbates the damage of the colon [25]. The activation immune cells and cytokines including ILs, and TNF-α regulate cytotoxicity of epithelial cells and enhance the immune response of the intestinal tissue [11]. T helper cell-1 (Th1) could mediate the immune response by releasing IL-2, Interferon-γ (IFN-γ), and TNF-α [26–28]. The increased concentration of TNF-α mediated by IL-9 performs negative effect on the function of intestinal barrier [29, 30]. Our results revealed the significant increase of *TNFα*, *IL-6* and *IL-1β* in control group. However, after HKL treatment, these pro-cytokines were decreased, which showed the significantly clinical function in adaptive immune response.

The effects of HKL on regulating immune response were further validated by transcriptomic analysis. A total of 670 DEGs (415 up-regulated and 255 down-regulated genes) were identified between HKL treated UC rats and water treated UC rats. Functional enrichment analysis suggested that these DEGs were mostly related with immune system, including “leukocyte mediated immunity”, “lymphocyte mediated immunity”, “immune response”, and “B cell mediated immunity”. The DEGs involved in these functions included *C3*, *Cbl*, *Cd8a*, *Cfb*, *Chga*, *Dennd1b*, *Igh-6*, *Ighg1*, *Pla2g1b*, *Pram1*, *RT1-CE3*, *RT1-T24-1*, *Ripk3* and *Tfrc*. Among these DEGs, *C3* and *Cfb* are complement components which are found being upregulated in UC in previous studies [31, 32]. Complements are potent innate immune defense factors involved in intestinal homeostasis and activation of complements have been reported to be involved in UC for a long time [33, 34]. In our study, *C3* and *Cfb* were downregulated by HKL at a 3.24-fold and 3.85-fold, respectively. In line with this, the downregulated DEGs were significantly enriched in the pathway of “Complement and coagulation cascades”. These results suggested HKL might regulate immune response for UC rats.

We performed KEGG enrichment analysis for upregulated genes and downregulated genes, respectively. The KEGG pathways of “PPAR signaling pathway”, “ECM-receptor interaction”, “calcium signaling pathway”, “cGMP-PKG signaling pathway”, and “cAMP signaling pathway” were significantly activated after HKL treatment, while the KEGG pathways of “Complement and coagulation cascades”, “IL-17 signaling pathway”, “cell adhesion molecules (CAMs)” were significantly suppressed. The proliferator-activated receptor γ (PPAR-γ) activation plays key role in regulation of inflammation and immune response in UC patients and the anti-inflammatory effects of 5-ASA in UC patients are mediated by PPARγ activation [35]. After treatment with HKL, the PPAR signaling pathway was significantly activated in this study, showing similar mechanism of 5-ASA. The pathways of “IL-17 signaling pathway” and “cell adhesion molecules (CAMs)” were significantly suppressed after HKL treatment in this study. IL-17 is a key mediator in the pathogenesis of intestinal inflammation [36]. It acts as a potent inflammatory interleukin that activates the expression of other pro-inflammatory cytokines [37]. In a previous study, Abdelmegid et al. proposed that gold nanoparticles could effectively targeted colonic tissue by reducing IL-17 [38]. The IL-17 signaling pathway was suppressed in this study, suggesting the promising effect of HKL on UC.

Further, we selected KEGG pathways that might be related with UC progression and built a PPI network among the DEGs enriched in these pathways. Dhcr7 (degree = 17), Cyp2e1 (degree = 14), Pnlip (degree = 12), Cpb1 (degree = 12) and Kng1 (degree) were the 5 nodes with the highest node degree. Dhcr7 encodes delta-7-sterol reductase, which is the ultimate enzyme of mammalian sterol biosynthesis that converts 7-dehydrocholesterol to cholesterol [39]. Recent study suggested that cholesterol metabolism plays important role in innate immune response [40]. It is shown that expression of Dhcr7 is reduced by macrophages and it might be a potential therapeutic target against pathogenic viruses [41]. Cyp2e1 encodes cytochrome P450 2E1 is a member of the cytochrome P450 mixed-function oxidase system. Previously, Yamamoto et al. demonstrated that CYP2E1 is activated in serum obtained from UC rats and could be used as a biomarker for evaluating UC [42]. In this study, Dhcr7 and Cyp2e1 were significantly downregulated by 1.39-fold and 3.71-fold after HKL treatment, indicating the role of HKL in relieving UC.

Metabolic abundance analysis provided another way to evaluate the therapeutic mechanisms. In a previous study, tricarboxylic acid (TCA)-trans-aconitate was found decreased in UC patients [43]. Besides, serum levels of 3-hydroxybutyrate and acetoacetate were found elevated in UC patients compared with controls [44, 45]. Low concentration of cysteine is also observed in UC patients, which is known as limiting substrates in the biosynthesis of glutathione [46]. Also, it is reported that significant variations TCA cycle-related molecules were observed in the sera of the UC patients [47]. In this study, after integrating of transcriptome and metabolite analysis, “steroid hormone biosynthesis”, “pyrimidine metabolism”, “purine metabolism”, and “glutathione metabolism” were altered in UC progression and might be the therapeutic targets of HKL. These results revealed correlation with previous studies and explored the underlying mechanism in HKL therapy. Further, the rarely reported significance of pyrimidine metabolism and purine metabolism provided the new therapeutic evidence. We also observed mineral absorption and bile secretion were enriched in metabolite and transcription integrative analysis. Previous reports indicated that vitamin and minerals supplements could be used for treating IBD [48–50]. Our study firstly showed mineral absorption might function in UC progression, which provided the treatment option in UC patients. In UC, the deficiency of passive absorption in the colonic tract could lead to the variation of bile acids pool [51]. Usually, inflammation often is associated with deficit bile acids malabsorption [52]. Our research revealed bile absorption played a role in UC pathology.

In conclusion, we investigated the promising effects of HKL on UC and its related molecular mechanism in TNBS-induced UC model. The results revealed that HKL could significantly reduce pro-inflammatory cytokines expression. Integrative analysis of transcriptomic and metabolomic profiling in water treated UC and HKL treated UC samples provided us the immune pathway might be the therapeutic targets of HKL. These results shed light on the clinical application of HKL. Further clinical trial research is needed to help demonstrating the pharmacology of HKL.

## MATERIALS AND METHODS

### Experimental rats

Specific pathogen free male Wistar rats (220-250g) were obtained from Xinjiang Medical University Animal Center, Urumqi, China and housed in the controlled condition with 25° C temperature and 70%-75% humidity. The rats were feed with standard diet before use. All the experiments were approved by the ethics committee of Xinjiang Medical University (Permit number: (IACUC20121122011).

### UC model establishment

After one week of acclimation, the UC model was established by using TNBS enema according to literature [53]. Briefly, Wistar rats were randomly divided in to 4 groups with 10 rats in each group: normal group, water-treated group, HKL-treated group and 5-ASA treated group. TNBS was used to induce UC. The rats were fasted for 24 h and anaesthetized by intraperitoneal injection of sodium pentobarbital (40 mg/kg). Then, TNBS (70 mg/kg) was dissolved in 50% ethanol, and the mixed solution was injected into the proximal end of the descending colon slowly using 3-mm enema tube. The rats were kept inverted vertical position for 30s to facilitate the diffuse distribution of TNBS solution in intestine. The rats in control group received injection of physiological saline. After 3 days, rats in each group were subjected for drug treatment.

### Treatment of rats

After 3-day of UC model establishment, UC rats in HKL group were clustered with HKL at dose of 1.8 g/kg/day. UC rats in 5-ASA group were treated with 5-ASA at a dose of 100 mg/kg/day. Rats in water treated group and control group were given sterilized water at 2 ml/day for 10 days. Weight, stool characters, mental state, hair and activity were recorded daily. Rats were sacrificed by decapitation under anesthesia after treatment for 3 days, 7 days and 10 days. Different parts of the colon were isolated including caecum and lymphoid for histology assay and immunohistochemistry analysis.

### Histological analysis

Histological analysis was assessed as described previously. Briefly, 8 um cryostat sections were picked up and dried. After fixing by formalin buffer, hematoxylin-eosin staining was introduced. Histological scores were quantified by two blinded researchers based on reference [54]. Amount of inflammation and extent of lesion were evaluated with a range from 0 to 3. Depth of the inflammation was assessed with a range from 0 to 4 to evaluate the damage and regeneration. Also, the scores were quantified as to the percentage of UC progress: 1-25%, 26-50%, 51-75% and 76-100%. Each section was scored to establish the grade and percentage (0-12 for inflammation and 0-16 for regeneration). Experiments were performed in triplicate.

### RNA extraction and qRT-PCR

Total RNA of colon tissues after treating for 3 days, 7 days and 10 days was extracted using TRIzol reagent (TaKaRa, Dalian, China) and was reverse transcribed using PrimeScript™ RT Master Mix (TaKaRa). qRT-PCR was performed using 2× Power SYBR green mix (Thermo Fisher Scientific, Waltham, MA, USA) on an ABI 7500 sequencer (Thermo Fisher Scientific). The primer sequences were listed in Table 1. Relative expression of genes was determined using 2^-ΔΔCt^ method using GAPDH as an internal reference. The gene expression was tested in three rats at each time points and each sample was tested in triplicate.

**Table 1:** The sequence of primers in qRT-PCR.

**Gene**	**Direction**	**Sequence (5’-3’)**
IL4	Forward	ACAAGGAACACCACGGAGAA
	Reverse	CAGACCGCTGACACCTCTACA
IL6	Forward	AAGAAAGACAAAGCCAGAGTC
	Reverse	CACAAACTGATATGCTTAGGC
IL10	Forward	AGAAGGACCAGCTGGACAACAT
	Reverse	CAAGTAACCCTTAAAGTCCTGCAGTA
IL1β	Forward	CCCTGCAGCTGGAGAGTGTGG
	Reverse	TGTGCTCTGCTTGAGAGGTGCT
TNFα	Forward	TCAGCCTCTTCTCATTCCTGC
	Reverse	TTGGTGGTTTGCTACGACGTG
GAPDH	Forward	AGACAGCCGCATCTTCTTGT
	Reverse	CTTGCCGTGGGTAGAGTCAT

### Western blot

Colon tissues after treating for 7 days was lysed in RIPA buffer (Beyotime Biotechnology, Shanghai, China) containing PMSF and centrifugated at 12000 × g for 10 min at 4° C. Protein concentration was determined by a bicinchoninic acid kit (PL212989, Thermo). Equal amounts of protein were separated by 10% SDS-PAGE and transferred to a PVDF membrane. The membranes were blocked with 5% non-fat milk and incubated with primary antibodies (IL-10, ab9969, Abcam, 1:500; IL-1β, 66737-1-Ig, Proteintech, 1:2000; IL-4, 66142-1-Ig, Proteintech, 1:1000; IL-6, 66146-1-1Ig, Proteintech, 1:1000; TNF-α, ab6671, Abcam, 1:1000; β-actin, Sc-47778, Santa Cruz, 1:5000) overnight at 4° C. The membranes were then incubated with goat anti-rabbit or goat anti-mouse (Jackson ImmunoResearch) for 1 h at room temperature. β-actin served as a loading control. Band intensity was determined using a chemiluminescent imaging system (Tanon, Shanghai, China), and ImageJ (NIH, Bethesda, MD, USA) was used for quantification.

### Transcriptomic profiling

Total RNA was extracted from colon tissues of UC rats in water group and HKL group (n = 3 in each group) at 7-day treatment using TRIzol® Reagent (Thermo Fisher, Waltham, MA, USA). The quality and quantity of total RNA were evaluated using Nanodrop 2000 (Agilent Technologies, Santa Clara, CA, USA). Independent cDNA libraries were constructed using Truseq™ RNA sample prep Kit (Illumina, San Diego, CA, USA) and the cDNA libraries were sequenced on Illumina Hiseq2500 (Illumina). The data could be assessed from NCBI SRA database with the accession number of PRJNA627528.

### Bioinformatics analysis of RNA sequencing data

The raw sequencing data were quantified using FastQC v0.11.4. Clean reads were obtained by removing adapter sequences and low-quality bases using cutadapt v1.16 (http://cutadapt.readthedocs.io/). Then, clean reads were aligned to reference genome of Rat (Rnor_6.0) using hisat v2.1.0 (https://ccb.jhu.edu/software/hisat2/index.shtml). The fragments per kilobase of transcript sequence per million base pairs sequence (FPKM) for each sample were estimated using Stringtie v1.3.3b (http://ccb.jhu.edu/software/stringtie/). Differential gene expression analysis was implemented using the edgeR (v 3.24, http://www.bioconductor.org/packages/release/bioc/html/edgeR.html) with the P < 0.05 and |log_2_ FC| >1. DEGs were visualized using in-house scripts of plot_scatter_exp (v1.1.0) and plot_volcano_exp (v1.1.0). GO and KEGG analyses of DEGs were conducted using in-house scripts of go_anot_exp (v1.4.0) and kegg_anot_exp (v1.4.0) respectively. GO-terms or KEGG pathways with adjusted P value < 0.05 were regarded as significant. PPIs among DEGs were predicted using STRING (http://string-db.org) database and a PPI network was visualized by Cytoscape (version 3.6.2).

### Metabolites extraction

Fecal samples of rats in water group and HKL group (n = 6 in each group) after treatment for 7 days were prepared in Eppendorf tube. After the addition of 1000 μL of extract solvent (acetonitrile-methanol-water, 2:2:1, containing internal standard 1 μg/mL), the samples were vortexed for 30 s, homogenized at 45 Hz for 4 min, and sonicated for 5 min in ice-water bath. The homogenate and sonicate circle were repeated for 3 times, followed by incubation at -20° C for 1 h and centrifugation at 1200 ×g and 4° C for 15 min. The resulting supernatants were transferred to LC-MS vials and stored at -80° C until the UHPLC-QE Orbitrap/MS analysis. Three QC samples were prepared by pooling aliquots of the supernatants from all the samples.

LC-MS/MS analyses were performed using an UHPLC system (Agilent Technologies) with a UPLC HSS T3 column (2.1 mm × 100 mm, 1.8 μm) coupled to Q Exactive (Orbitrap MS, Thermo). The mobile phase A was 0.1% formic acid in water for positive, and 5 mmol/L ammonium acetate in water for negative, and the mobile phase B was acetonitrile. The elution gradient was set as follows: 0 min, 1% B; 1 min, 1% B; 8 min, 99% B; 10 min, 99% B; 10.1 min, 1% B; 12 min, 1% B. The flow rate was 0.5 mL/min. The injection volume was 2 μL. The QE mass spectrometer was used for its ability to acquire MS/MS spectra on an information-dependent basis during an LC/MS experiment. In this mode, the acquisition software (Xcalibur 4.0.27, Thermo Fisher) continuously evaluates the full scan survey MS data as it collects and triggers the acquisition of MS/MS spectra depending on preselected criteria.

### Data preprocessing and annotation

The raw data were converted to the mzXML format using ProteoWizard, and processed by MAPS software (version 1.0). The preprocessing results generated a data matrix that consisted of the retention time (RT), massto-charge ratio (m/z) values, and peak intensity. The identification of metabolites was conducted by in-house MS2 database based on RT, m/z and peak intensity. Principal component analysis plots were used to evaluate data quality. Important metabolites were selected according to VIP score derived from applying partial least squares discriminant analysis or orthogonal partial least squares discriminant analysis. Differential metabolites were identified based on criteria of VIP > 1, P < 0.05 and |log_2_FC| > 0.565. Metabolic pathways were linked by differential metabolites according to KEGG database.

### Integrative metabolic and transcriptomic profiling data

The correlation between DEGs and differential metabolites was calculated by Spearman rank correlation analysis. Correlation coefficient (Q-value) < 0.05 was regarded as significance level. The overlapped KEGG pathways of DEGs and differential metabolites were obtained based on criteria of num_overlapping_genes > 0, num_overlapping_metabolites > 0 and P < 0.05.

### Statistical analysis

Statistical analyses were performed by GraphPad Prism 5 and data were showed as mean ± standard deviation (SD). Student’s t test with three repeats or analysis of variance (ANOVA) with Bonferronic post-hoc analysis were used to analyze the differences among groups when appropriate. *P* < 0.05 was regarded as statistically significant.

## Supplementary Material

Supplementary Table 1

Supplementary Table 2

Supplementary Table 3

Supplementary Table 4

Supplementary Table 5
